# A Putative Guanosine Triphosphate Cyclohydrolase I Named CaGCH1 Is Involved in Hyphal Branching and Fruiting Development in *Cyclocybe aegerita*

**DOI:** 10.3389/fmicb.2022.870658

**Published:** 2022-04-22

**Authors:** Nan Tao, Bopu Cheng, Hongmei Chai, Xianghua Cui, Yuanhao Ma, Jinping Yan, Yongchang Zhao, Weimin Chen

**Affiliations:** ^1^Biotechnology and Germplasm Resources Institute, Yunnan Academy of Agricultural Sciences, Kunming, China; ^2^Yunnan Provincial Key Lab of Agricultural Biotechnology, Kunming, China; ^3^Key Lab of Southwestern Crop Gene Resources and Germplasm Innovation, Ministry of Agriculture, Kunming, China; ^4^College of Life Sciences, Southwest Forestry University, Kunming, China; ^5^Life Science and Technology College, Kunming University of Science and Technology, Kunming, China

**Keywords:** GCH1, fruiting body development, *Cyclocybe aegerita*, RNAi, hyphal branch

## Abstract

Guanosine triphosphate (GTP) cyclohydrolase I (GCH1) is the limiting enzyme of the tetrahydrobiopterin (BH4) synthesis pathway. The disruption of *gch1* gene may cause conditional lethality due to folic acid auxotrophy in microorganisms, although the function of *gch1* in basidiomycetes has not been deciphered so far. In the present study, *gch1* expression in *Cyclocybe aegerita* (*cagch1*) was downregulated using the RNAi method, which resulted in growth retardation in both solid and liquid medium, with the hyphal tips exhibiting increased branching compared to that in the wild strain. The development of fruiting bodies in the mutant strains was significantly blocked, and there were short and bottle-shaped stipes. The transcriptional profile revealed that the genes of the MAPK pathway may be involved in the regulation of these effects caused by *cagch1* knockdown, which provided an opportunity to study the role of *gch1* in the development process of basidiomycetes.

## Introduction

Guanosine triphosphate (GTP) cyclohydrolase I (GCH1; EC 3.5.4.16) is the first enzyme to act in the biosynthesis of tetrahydrobiopterin (BH4). GCH1 catalyzes the conversion of GTP into dihydroneopterin triphosphate and formic acid (Nardese et al., 1996), and this reaction is the rate-limiting step in the *de novo* synthesis of BH4 in plants and microorganisms (Burg and Brown, 1968). Although bacteria, fungi, and plants can synthesize tetrahydrofolic acid; mammals consume folates in the form of vitamins in food. BH4 is crucial to invertebrates as it serves as a coenzyme of aromatic amino acid hydroxylase (Werner et al., 2011) and a cofactor of nitric oxide synthase (NOS) (Gorren and Mayer, 2002), in addition to being an essential regulatory factor of melanin biosynthesis (Chen et al., 2015) and a component of the synthesis process of various hormones and neurotransmitters (Werner et al., 2011). BH4 also serves as a cofactor for a variety of inflammatory factors and neurotransmitters (Latremoliere et al., 2015). In addition to being crucial for BH4 synthesis, GCH1 is reported to play an important role in the pathogenesis of neuropathological pain (Kim et al., 2009), Parkinson’s disease (Xu et al., 2017), diabetes (Wang et al., 2020), and cardiovascular diseases (Chuaiphichai et al., 2014).

Guanosine triphosphate cyclohydrolase I GCH1 is reported in various microorganisms, including *Escherichia coli* (Katzenmeier et al., 1990), *Saccharomyces cerevisiae* (Nardese et al., 1996), *Nocardia* sp. (He et al., 2004), and *Rickettsia monacensis* (Bodnar et al., 2020). The comparison of sequences from different organisms has revealed that the enzyme was highly conserved throughout the evolutionary process. The functional analysis of GCH1 has been focused mostly on the relationship between the enzyme and the folate content in organisms, such as folate enrichment by expressing the GCH1 gene in plants (Hossain et al., 2004; Storozhenko et al., 2007; Naqvi et al., 2009; Nunes et al., 2009; Blancquaert et al., 2015). So far, no one has described the effect of this enzyme on the development of the host organism. Furthermore, little is known about the function of GCHI in fungi. According to a report, the disruption of GCH1 in *S. cerevisiae* leads to conditional lethality due to folic acid auxotrophy (Nardese et al., 1996).

*Cyclocybe aegerita* is a delicious species cultivated in Asia (Zhang et al., 2015). The species has a short life cycle and complex development modes, such as monokaryon fruiting, which renders this species important as a model organism in genetics research (Labarère and Noël, 1992). In the present study, a gene encoding GCH1 named *cagch1* was isolated from *C. aegerita* YSG, and its genome sequence was cloned, followed by comparing the deduced protein sequences with those of the other species for homology analysis. Using the RNAi method, the morphologies of the mycelium, as well as the young and mature fruiting bodies, were compared between the wild stain and the GCH1-mutant strain at various developmental stages. The findings of the current study would provide novel insights into the role of GCH1 in basidiomycetes development.

## Materials and Methods

### Strains and Culture Conditions

*Cyclocybe aegerita* dikaryotic strain YSG deposited in our laboratory was used in the transformation experiments. The mycelia were cultured at 25°C on a yeast extract peptone dextrose (YPD) medium plate (0.2% yeast extract, 0.2% peptone, 2% dextrose, and 1.5% agar) followed by culturing in the sawdust medium (64% hardwood sawdust, 15% wheat bran, 1% gypsum, and 20% cottonseed shells in 50% water) to produce fruiting bodies. The protoplast regeneration medium contained 205 g of sucrose, 2 g of tryptone, 2 g of yeast extract, and 10 g of agar per liter of dH_2_O. The DH5α strain of *E. coli*, to be used for plasmid amplification, was cultured at 37°C in Luria–Bertani (LB) medium containing 100 μg/ml of ampicillin. In order to conduct the growth rate test, equal amounts of mycelia were inoculated in both solid and liquid YPD media for the evaluation of colony diameter and biomass, respectively.

### *Cagch1* Cloning and Sequencing

*Cyclocybe aegerita* YSG strain was cultured in YPD medium at 25°C in the dark for 8 days. The genomic DNA was extracted from the mycelia obtained from this culture of *C. aegerita* using the Fungal gDNA Isolation Kit (Biomiga, United States). Total RNA was extracted from the fungus using the RNAiso Plus in accordance with the manufacturer’s instruction (TaKaRa, Dalian, China). The complete genomic DNA and cDNA products of the *cagch1* gene were amplified in a polymerase chain reaction (PCR) using the primer pair gch1F and gch1R based on the genomic information of *C. aegerita* monokaryotic strain YSG. The amplified products/fragments were then cloned into the pMD19-T vector (TaKaRa, Dalian, China) and sent for sequencing (Tsingke Biotechnology Co., Ltd.).

### RNAi Vector Construction and the Transformation of *Cyclocybe aegerita*

The transformation vector pAGH (given by Dr. Zhang, Kunming University of Science and Technology) was used for constructing the RNAi vector using the method described in a previous report (Zhang et al., 2021). The plasmid pAGH comprised the *C. aegerita actin* promoter, the glyceraldehyde-3-phosphate dehydrogenase (*gpd*) gene promoter, the CaMV35S terminator, the hygromycin B phosphotransferase gene, and the *gpd* promoter from *Ustilago maydis*. A 487-bp antisense fragment, amplified using primer Gch1-1F and Gch1-1R (Supplementary Table 1), of the *cagch1* cDNA was also inserted into the *Eco*RV enzyme site. All fragments were sequentially ligated into the pAGH vector using the ClonExpress MultiS One Step Cloning Kit (Vazyme, Nanjing, China) in accordance with the manufacturer’s instructions.

The transformation was performed by following the PEG-CaCl_2_ protocol described in previous reports (Tao et al., 2020) with a few modifications. In brief, the protoplasts were prepared by digesting the *C. aegerita* mycelia from the liquid culture with a lysing enzyme solution containing 1% cellulase R-10 (Japan), 1% Lywallzyme, and 1% lysing enzymes from *Trichoderma harzianum* (Sigma-Aldrich) in 0.6 M mannitol buffer as the osmotic stabilizer. The obtained protoplasts were washed in the STC solution [1.2 M sorbitol, 10 mM CaCl2, and 50 mM Tris–HCl (pH 7.0)] and adjusted to a final concentration of 1 × 10^8^ protoplasts ml^–1^. Subsequently, a volume of 100 μl of protoplasts was mixed with 1 μg of plasmid DNA digested with the *Kpn*I enzyme and the reaction was allowed to occur for 30 min on ice. Afterward, 1 ml of the PTC solution (40% w/v polyethylene glycol 4,000, 60 mM CaCl2, and 1.2 M sorbitol) was added to the mixture, followed by incubation for 30 min at room temperature. Finally, the cells were added to 10 ml of molten YPD regeneration medium and then poured on plates. After 5 days of incubation at 25°C, 10 mL of molten YPD medium containing 150 μg/ml hygromycin B was poured over the plates for the screening of transformants.

### Transformant Verification and Quantitative Real-Time Polymerase Chain Reaction

The colonies resistant to hygromycin B were verified using the primer pair adhF and adhR targeted to the flanking region of the inserted fragment. Quantitative PCR (qPCR) was performed in the CFX96 Real-Time PCR detection system (Bio-Rad, Hercules, CA, United States). The PCR reaction was performed in a total volume of 20 μL using the iTaq™ Universal SYBR Green Supermix in accordance with the manufacturer’s instructions (Bio-Rad). The thermal cycling parameters for the PCR reactions were as follows: 20 s at 95°C for initial polymerase activation, followed by 40 cycles of 5 s at 95°C, and 30 s at 60°C. The primers used are listed in Supplementary Table 1. Amplification of each sample was performed in triplicate. Glyceraldehyde-3-phosphate dehydrogenase (GPD) was used as the reference gene for the normalization of the qPCR data. The relative expression levels were calculated using the 2^–ΔΔCT^ method.

### Transcriptome Analysis

Total RNA was isolated from the mature YSG fruiting bodies as well as the transformants T5, T10, and T11. The corresponding transcriptomic data were obtained from six cDNA libraries sequenced on an Illumina sequencing platform at the Beijing Genome Institute (BGI, Shenzhen, China). The differentially expressed genes (DEGs) were classified into GO and Kyoto Encyclopedia of Genes and Genomes (KEGG) pathways.

## Results

### Sequence Characterization for *Cagch1*

**FIGURE 1:**
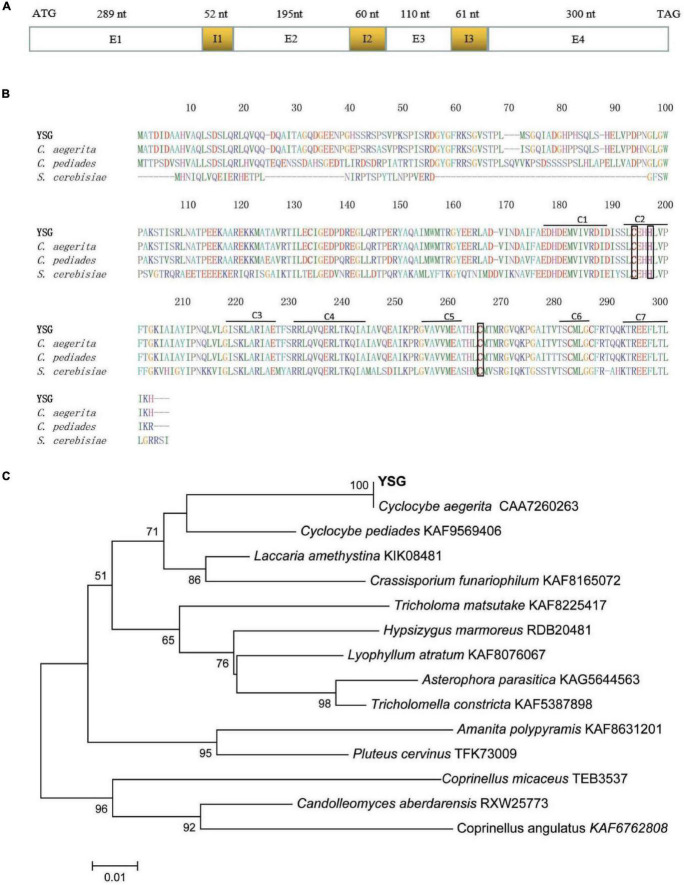
**(A)** Sequence structure of *cagch1*. **(B)** The characterization of the sequence of CaGCH1 (YSG) and its comparison with the sequences from other strains including *C. aegerita* (Accession Number CAA7260263), *C. pediades* (Accession Number KAF9569406), and *S. cerevisiae* (Accession Number P51601). **(C)** The phylogenetic tree for CaGCH1 with other GCH1 from basidiomycetes.

On the basis of the genome annotation and sequence analysis, gDNA and cDNA were employed as templates for the amplification of the gene encoding CaGCH1 (the *cagch1* gene), and the amplification products were sequenced and analyzed. The gDNA sequence of *cagch1* was 1,067 bp in length and contained 3 introns and 4 exons (Accession Number OL303985). The cDNA of *cagch1* was 894 bp in length and contained 297 amino acids (Figure 1A).

The protein sequences were aligned using ClustalW (soft package included in Mega 6) (Tamura et al., 2013) and the result revealed that the CaGCH1 protein shared a high level of similarity with the GCH1 proteins of *C. aegerita* (Accession Number CAA7260263), *Cyclocybe pediades* (Accession Number KAF9569406), and *S. cerevisiae* (Accession Number P51601). All four proteins contained seven conserved regions (C1–7) and three Zn^2+^ binding residues (Figure 1B). The phylogenetic analysis of the GCH1 protein sequences was performed with MEGA6 software using neighbor-joining, minimum evolution (Tamura et al., 2013), which indicated that CaGCH1 was the closest to that of the *Cyclocybe* species (previously used genus name *Agrocybe*) (Figure 1C).

### Transformant Verification and Expression of *Cagch1*

**FIGURE 2:**
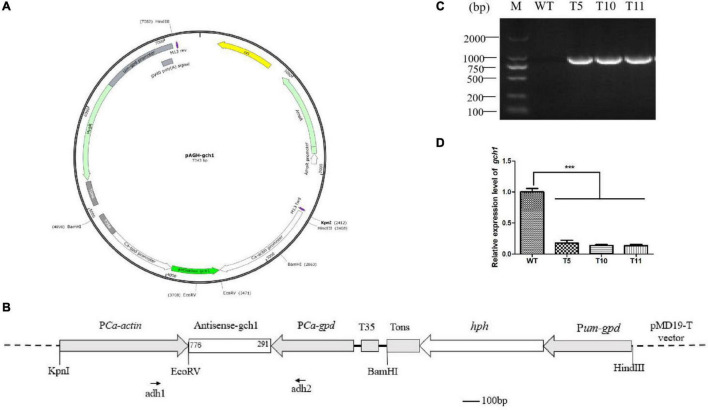
**(A)** Structure of the pAGH-gch1 plasmid. **(B)** Diagram of the RNAi vector construction. The antisense fragment of *cagch1* was inserted in the site between the promoters *actin* and *gpd*. The primer pair of adh1 and adh2 was used for the verification of the transformants. **(C)** The verification of transformants. **(D)** The expression analysis of *cagch1*. Asterisks (***) indicate the data that differed significantly based on *p* < 0.0001 (*t*-test) as the significance threshold.

In order to study the effect of *cagch1* on the developmental process, the silence transformants were obtained by constructing a dual RNAi plasmid (Figure 2A). After the screening of transformants on the YPD medium containing hygromycin B, the specific primer pair adhF and adhR targeted to the *actin* and *gpd* promoter regions was used for confirming the positive transformants through PCR amplification (Figure 2B). Finally, correct PCR products were detected in three strains T5, T10, and T11 (Figure 2C).

Mycelia were collected from WT, T5, T10, and T11 and subjected to total RNA extraction. The cDNA obtained after the reverse transcription of the extracted RNA was used for quantifying the expression of *cagch1*. It was observed that the expression level of *cagch1* in all three transformants had decreased to a low level – approximately 17.63% in T5, 13.87% in T10, and 13.53% in T11 compared to that in the WT strain (Figure 2D).

### Effect of *Cagch1* on Mycelial Growth and Morphology

**FIGURE 3:**
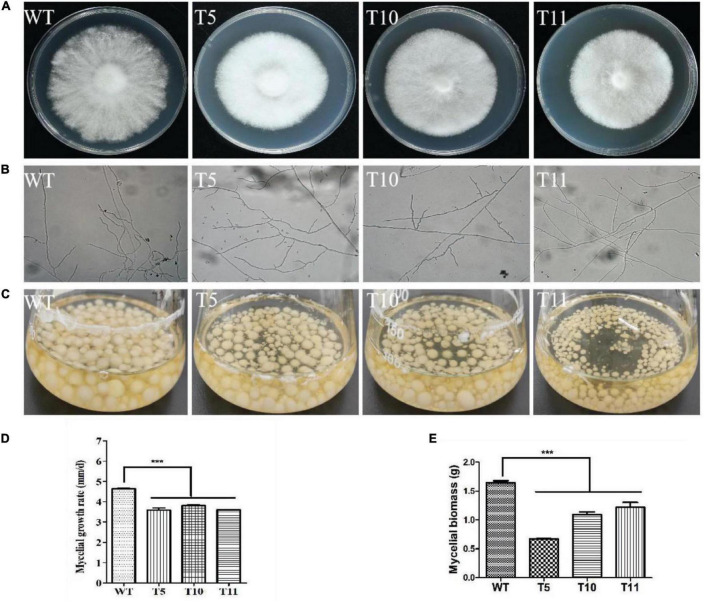
Morphology and growth characteristics of WT, T5, T10, and T11 strains. **(A)** Colony morphology. **(B)** Hyphal branch. **(C)** Culture in liquid. **(D)** Mycelial growth. **(E)** Mycelial biomass. Asterisks (***) indicate the data that differed significantly based on *p* < 0.0001 (*t*-test) as the significance threshold.

After 7 days of culture on YPD plates, the transformants colonies appeared denser with smooth edges than WT colonies (Figure 3A). The colony diameter was measured for growth rate analysis, and it was observed that the WT strains spread faster than the transformants (Figure 3D). The microscopic observation revealed that transformants exhibited relatively greater branching in the hyphal tips growing on the cover glass (Figure 3B).

The equal mycelium block was cultured in the liquid YPD medium under shaking conditions of 180 rpm/min for 7 days (Figure 3C). It was observed that the mycelial biomass of all three transformants was decreased significantly compared to the WT strain, with the mycelium weight of the T5 strain approximately one-half that of the WT strain (Figure 3E).

### Effect of *Cagch1* on the Fruiting Bodies

**FIGURE 4:**
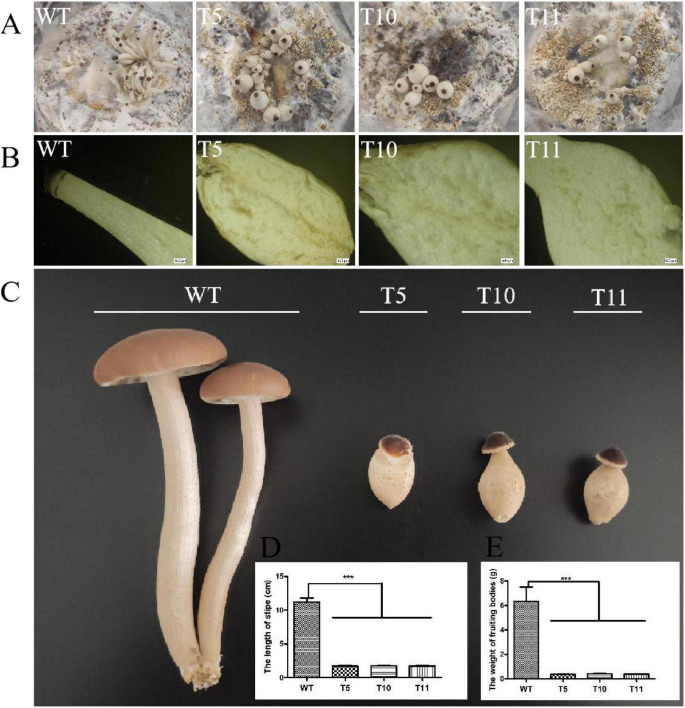
Morphology of the fruiting bodies in WT, T5, T10, and T11. **(A)** Young fruiting bodies. **(B)** Longitudinal section image of stipes. Bar, 500 μm. **(C)** Mature fruiting bodies. **(D)** The length of stipes. **(E)** The weight of fruiting bodies. Asterisks (***) indicate the data that differed significantly based on *p* < 0.0001 (*t*-test) as the significance threshold.

The WT strain and the three mutant strains were cultured in sawdust bags at 25°C for 40 days to investigate the potential effect of *cagch1* on the fruiting bodies of *C. aegerita*. The development of the transformant strains was observed to be blocked, and there were bottle-shaped stipes (Figure 4A). The longitudinal section of young fruiting bodies revealed that the stipes of the mutant strains comprised flocculent flesh relative to the compact flesh of the WT strain (Figure 4B). At the late stage, the stipe elongation in the mutant strain was significantly inhibited, and the length and the weight of fresh fruiting bodies were approximately one-sixth and one-fifteenth that of the wild type, respectively (Figures 4C–E). Moreover, the morphologies of all strains were stable in the subsequent two culture processes.

**FIGURE 5:**
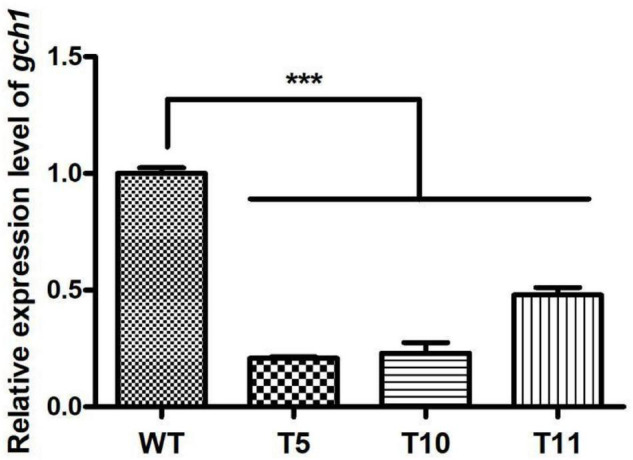
The expression level of cagch1 in mature fruiting bodies. Asterisks (***) indicate the data that differed significantly based on *p* < 0.0001 (*t*-test) as the significance threshold.

Furthermore, the expression level of *cagch1* in the mature fruiting bodies was evaluated using the qPCR method. As depicted in Figure 5, the expression level of *cagch1* in the three transformants exhibited different degrees of decrease—approximately 21.83% in T5, 23% in T10, and 48% in T11 compared to that in the WT strain (Figure 5).

### Transcriptional Profile of the RNAi Strain

In order to evaluate the effect of *cagch1* knockdown on the expression levels of different genes, the expression difference in the mating bodies of WT was compared to those of each of the three transgenic strains using the transcriptional profile analysis (PRJNA813015).

**FIGURE 6:**
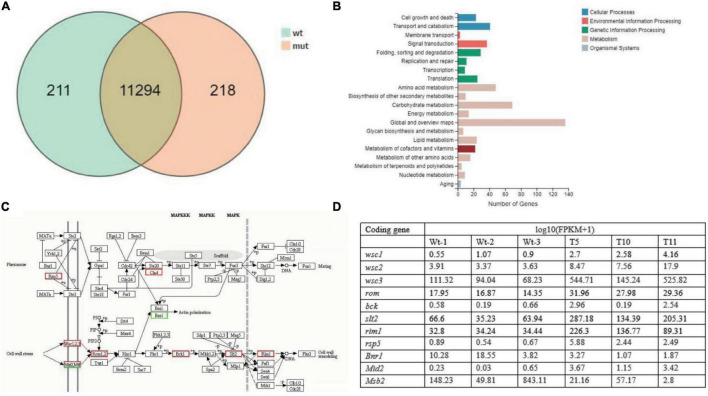
The number of differentially expressed genes (DEGs). **(A)** Venn diagram depicting the number of DEGs between WT and transformant strains (mut). **(B)** Kyoto Encyclopedia of Genes and Genomes (KEGG) classification of the identified DEGs. **(C)** The putative pathway of signal transduction deduced in the KEGG analysis. **(D)** The expression levels of genes in the MAPK pathway.

A total of 11,723 differentially expressed unigenes (DEGs) were identified, including 211 and 218 special genes in the WT fruiting bodies and in the mutant fruiting bodies, respectively (Figure 6A). Furthermore, the 544 DEGs involved in the KEGG metabolic pathway were categorized into five major types comprising 20 categories, among which a few pathways were associated with mycelial growth and development, such as cell wall remodeling in signal transduction pathway and polysaccharide anabolism pathway (Figure 6B). Among these 544 genes, most annotated in the MAPK pathway (Figure 6C) and upregulated in the mutant strains were possibly involved in cell wall remodeling and the mating process (Figure 6D).

## Discussion

Guanosine triphosphate cyclohydrolase I plays an important role in the biosynthetic pathway of various pteridines, including folic acid derivatives and tetrahydrobiopterin, where it serves as the enzyme cofactor for the one-carbon transfer reaction and the hydroxylation reaction of aromatic amino acids, respectively (Nardese et al., 1996). GCH1 is also involved in the production of nitric acid and, therefore, regulates several physiological processes via various molecular pathways (Tayeh and Marletta, 1989). In order to investigate the potential function of GCH1 in *C. aegerita*, the *cagch1* gene was downregulated by constructing an RNAi system for *cagch1*. The results revealed that the transformants exhibited variable morphology during different developmental stages.

As an important functional enzyme, GCH1 contains amino acid sequences that are conserved in *Homo sapiens*, plants, and microorganisms (He et al., 2004; Liang et al., 2019; Bodnar et al., 2020). In particular, certain key residues for binding GTP or coordinating with the zinc ion, such as HH, LSK, and QE, present in GCH1 have been identified in CaGCH1 motifs C2, C3, and C4. Similar to the results reported in previous studies (He et al., 2004; Liang et al., 2019; Bodnar et al., 2020), in the present study, the amino acid residues of CaGCH1 were observed to be highly conserved in the downstream region compared to those of other species. These sequence characteristics provided a basis for the identification of GCH1, which is usually not annotated in the genome database.

The RNAi system was successfully constructed in *C. aegerita* using a dual promoter. The targeted gene cagch1 was evidently downregulated during the development of transformants. In a previous study on *Ganoderma lucidum*, four gene silencing constructs, namely, hairpin, sense, antisense, and dual promoter constructs, were introduced into the strain to be evaluated, with the dual promoter silencing vector demonstrating the highest rates of target gene silencing (Mu et al., 2012). Although the hairpin method has been used frequently for gene silencing in previous studies, the dual promoter method is simpler and more efficient in constructing the RNAi system in basidiomycetes.

The studies investigating the function of GCH1 in microorganisms are scarce. A study reported that the disruption of GCH1 in *S. cerevisiae* leads to conditional lethality due to the obstruction of folinic acid synthesis (Nardese et al., 1996). Another study reported that the important role of GCH1 in various physiological processes renders it suitable as a potential anti-infective drug target (Schüssler et al., 2019), which was confirmed when the inhibitors of tetrahydrofolate biosynthesis were successfully able to control a wide variety of microbial and protozoan infections (Swarbrick et al., 2008). However, the function of GCH1 in basidiomycetes has not been investigated so far. In the present study, the expression of *cagch1* in *C. aegerita* was downregulated, and it was observed that the transformants differed from the wild-type strains in terms of mycelial branching, biomass, and fruiting bodies. The growth of the transformants was evidently inhibited in both solid and liquid media and the mycelium exhibited greater branching. In a previous study on *G. lucidum*, the gene encoding nicotinamide adenine dinucleotide phosphate oxidases (Nox) was reported to play an important role in hyphal branching and fruiting body development through the regulation of the Ca^2+^ signal pathway (Mu et al., 2014). Another study indicated that a cross-talk between Ca^2+^ and reactive oxygen species (ROS) regulates hyphal branching (Gao et al., 2018).

As cofactors of NOS, NADPH, FAD, BH4, and FMN are involved in the production of nitric oxide (NO) (Song et al., 2000). In fungi, NO is involved in the regulation of various developmental processes, including sporulation, spore germination (Maier et al., 2001; Gong et al., 2007), and fruiting body production (Song et al., 2000). When the underlying mechanism was investigated, the involvement of cGMP, MAPK, and light signaling pathways was revealed (Barhoom and Sharon, 2004; Vieira et al., 2009). In the present study, most of the genes that had been annotated in the mating process and cell wall remodeling in the MAPK pathway were upregulated in the mutant strains, which suggested that these genes could be induced by the products associated with CaGCH1 for participating in the developmental process.

## Conclusion

A putative gene encoding the GCH1 protein, named *CaGCH1*, was cloned and identified in the basidiomycetes *C. aegerita*. The silencing of *cagch1* resulted in the retardation of development, including the growth and the following morphological characteristics: (1) the growth rate of the mutants was decreased; (2) the hyphal tips of the mutants exhibited greater branching; and (3) the fruiting bodies in the mutants had short and bottle-shaped stipes. The transcriptomic analysis revealed that the regulation of the MAPK pathway by the knockdown of *cagch1* could be involved in the development of fruiting bodies. Further comprehensive analysis of the function of *cagch1* would improve the understanding of its role in the development process in basidiomycetes.

## Data Availability Statement

The data presented in the study are deposited in the NCBI repository, accession number PRJNA813015.

## Author Contributions

WC and YZ conceived and designed the experiments. NT, BC, and JY carried out the transformation and screening. NT, BC, and YM investigated the growth and microscopic observation. HC, BC, and XC performed the RT-PCR experiments and transcriptome analysis. WC, NT, and YZ analyzed all data. WC and NT wrote original draft manuscript. All authors have read and agreed to the final version of the manuscript.

## Conflict of Interest

The authors declare that the research was conducted in the absence of any commercial or financial relationships that could be construed as a potential conflict of interest.

## Publisher’s Note

All claims expressed in this article are solely those of the authors and do not necessarily represent those of their affiliated organizations, or those of the publisher, the editors and the reviewers. Any product that may be evaluated in this article, or claim that may be made by its manufacturer, is not guaranteed or endorsed by the publisher.
