# Supplementary material for: A Putative Guanosine Triphosphate Cyclohydrolase I Named CaGCH1 Is Involved in Hyphal Branching and Fruiting Development in Cyclocybe aegerita
Source: Front Microbiol. 2022 Apr 22;13:870658. doi: 10.3389/fmicb.2022.870658 (PMC9076582; doi:10.3389/fmicb.2022.870658)
Supplement: Supplementary file 1 [file Table_1.DOCX]

**Table 1.** Primers used in this study.

| Primer | Sequence (5’ to 3’) | Description |
| --- | --- | --- |
| gch1F | taacgaataatagccgatatcATGGCCACCGACATCGACG | Amplification for the whole length of *cagch1* |
| gch1R | cggtcggcatctacgatatcTTAGATGTCTGGCTTTTGAACCC |  |
| Gch1-1F | TAACGAATAATAGCCGATATCGCACAAATGCGTGGCCTC | Amplification for antisense fragment of *cagch1* |
| Gch1-1R | ATCTCTCGTTTACGGCGCAAGTCGACCATTTCTCGTTTG |  |
| Adh1 | CACCGTAACGAATAATAGCCG | Verification of transformants |
| Adh2 | TGCGTGATTGTGATTGGC |  |
| Gch qF | CGATTATGTGGATGACTCGG | Detection for ca*gch1* expression level |
| Gch qR | AAGACGCTCCTGAACCTGTAG |  |
| Gpd qF | TCATCAATGGCAAGCCTG | Detection for *gpd* expression level |
| Gpd qR | CCAAGTTCACACCACAGACG |  |
